# Ingestion of Non-digestible Carbohydrates From Plant-Source Foods and Decreased Risk of Colorectal Cancer: A Review on the Biological Effects and the Mechanisms of Action

**DOI:** 10.3389/fnut.2019.00072

**Published:** 2019-05-15

**Authors:** Samira Bernardino Ramos do Prado, Victor Costa Castro-Alves, Gabrielle Fernandez Ferreira, João Paulo Fabi

**Affiliations:** ^1^Department of Food Science and Experimental Nutrition, School of Pharmaceutical Sciences, University of São Paulo, São Paulo, Brazil; ^2^Food Research Center (FoRC), CEPID-FAPESP (Research, Innovation and Dissemination Centers, São Paulo Research Foundation), São Paulo, Brazil; ^3^Food and Nutrition Research Center (NAPAN), University of São Paulo, São Paulo, Brazil

**Keywords:** colorectal cancer, dietary fiber, fermentation, galectin-3, non-digestible carbohydrates, pattern recognition receptors, pectin, tool-like receptors

## Abstract

The hypothesis that links the increase in the intake of plant-source foods to a decrease in colorectal cancer (CRC) risk has almost 50 years. Nowadays, systematic reviews and meta-analysis of case-control and cohort studies confirmed the association between dietary patterns and CRC risk, in which the non-digestible carbohydrates (NDC) from plant-source foods are known to play beneficial effects. However, the mechanisms behind the physicochemical properties and biological effects induced by NDC on the decrease of CRC development and progression remain not fully understood. NDC from plant-source foods consist mainly of complex carbohydrates from plant cell wall including pectin and hemicellulose, which vary among foods in structure and in composition, therefore in both physicochemical properties and biological effects. In the present review, we highlighted the mechanisms and described the recent findings showing how these complex NDC from plant-source foods are related to a decrease in CRC risk through induction of both physicochemical effects in the gastrointestinal tract, fermentation-related effects, and direct effects resulting from the interaction between NDC and cellular components including toll-like receptors and galectin-3. Studies support that the definition of the structure-function relationship—especially regarding the fermentation-related effects of NDC, as well as the direct effects of these complex carbohydrates in cells—is crucial for understanding the possible NDC anticancer effects. The dietary recommendations for the intake of NDC are usually quantitative, describing a defined amount of intake per day. However, as NDC from plant-source foods can exert effects that vary widely according to the NDC structure, the dietary recommendations for the intake of NDC plant-source foods are expected to change from a quantitative to a qualitative perspective in the next few years, as occurred for lipid recommendations. Thus, further studies are necessary to define whether specific and well-characterized NDC from plant-source foods induce beneficial effects related to a decrease in CRC risk, thereby improving nutritional recommendations of healthy individuals and CRC patients.

## Relationship Between the Intake of Plant-Source Foods and Decrease in Colorectal Cancer Risk

Cancer is one of the leading cause of death globally. Around one-third of cancer-related death are mostly connected to behavioral and dietary habits including tobacco and alcohol use, lack of physical activity, high body mass index, and low intake of fruits and vegetables (1). Colorectal cancer (CRC) is known to be associated mainly with dietary patterns of the so-called western lifestyle. The incidence of CRC is higher in developing countries and this incidence is increasing fast in both low- and middle-income countries. This is mainly due to a shift in dietary patterns toward a decreased intake of plant-source foods and an increased intake of fat, sugar and animal-source foods (2). Despite CRC is the second most diagnosed type of cancer in men and the third in women, as well as the third leading cause of all cancer death, only <10% of CRC arise from inherited syndromes (3, 4). Thus, studies have systematically pointed out that tackling modifiable risk factors, specially changing the dietary patterns, can substantially reduce CRC-related deaths (5–10).

Recently, a prospective longitudinal study revealed that a dietary pattern characterized by the high intake of plant-source foods is associated to a delayed CRC risk up to 10 years (11). Systematic reviews and meta-analysis of case-control and cohort studies also reported an inverse association between the intake of plant-source foods and CRC risk (12–14). Besides scientific data, the traditional knowledge suggests the intake of plant-source foods as adjuvant treatment against CRC (15). As plant-source foods contain relatively high amounts of biologically active molecules, such as polyphenols and non-digestible carbohydrates (NDC), the adoption of specific nutritional interventions using fruits and vegetables has been taken into consideration to assist cancer therapies (16). Furthermore, the increased ingestion of dietary fiber from plant-source foods, which are composed mainly by the NDC that constitute the plant cell wall, is known for a long time to play a pivotal role in the reduction of CRC risk (17–20).

Although NDC are resistant to digestion by human enzymes, these carbohydrates are not a static collection of food components that pass through the gastrointestinal tract without inducing biological effects. Instead, NDC modulate nutrient absorption through binding to organic molecules that induce indirect biological effects acting as substrate for colonic fermentation by the gut microbiota (21). Furthermore, recent efforts have focused on exploring the direct interaction between NDC and CRC cells that will be described further in this review. However, the composition and chemical structure of NDC may vary depending of plant species and tissues, thereby resulting in great heterogeneity of structure with variability in composition and branching pattern. Thus, although NDC from plant-source foods share common patterns and biological functions, the ingestion of these food components that have great variation in size and structure will result in structure-dependent properties and therefore diverse biological effects. In this review, we will describe some known mechanisms through which NDC from plant-source foods induce beneficial health effects that relate to a decrease in CRC risk.

## Structure of Non-Digestible Carbohydrates from Plant-Source Foods

**Figure 1:**
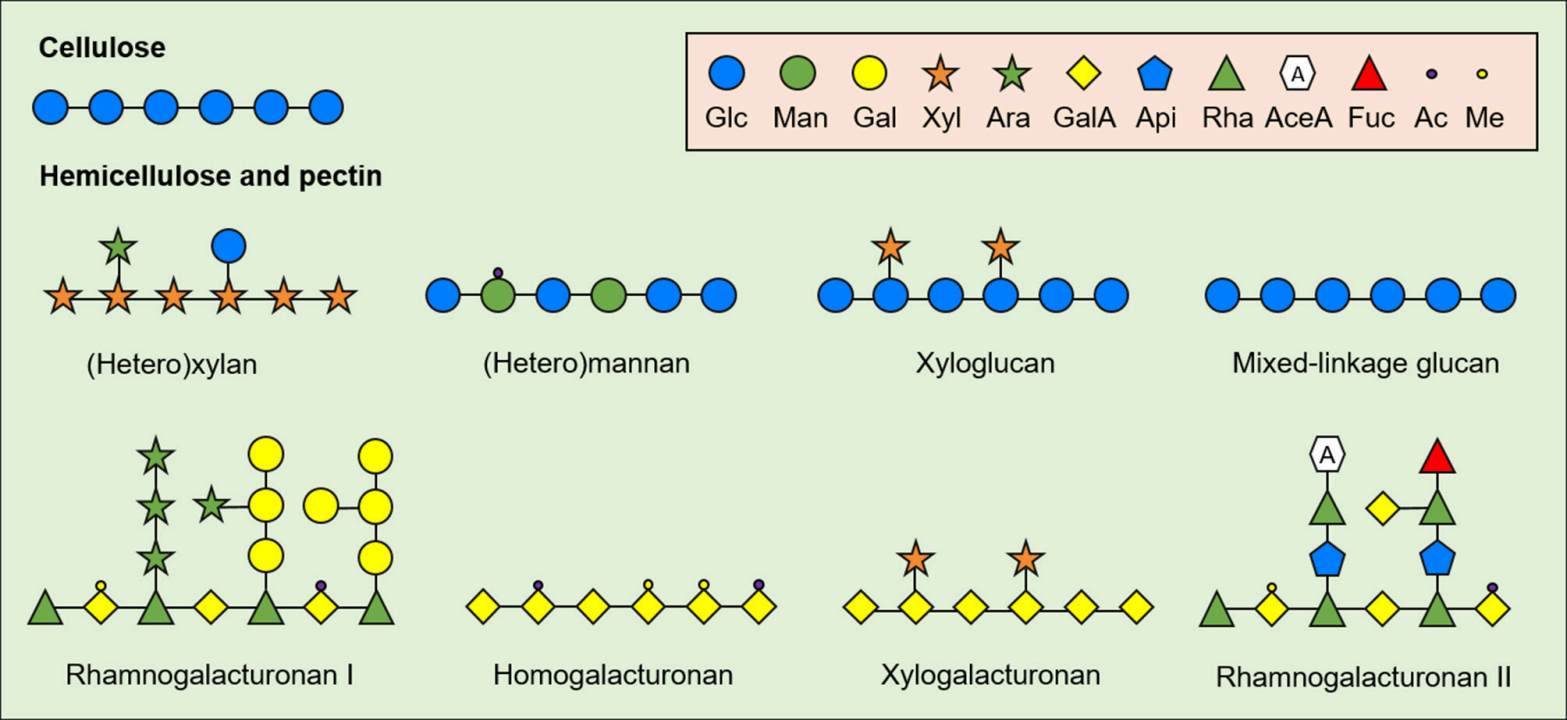
General structure of plant cell wall-derived non-digestible carbohydrates (NDC). Cell-wall derived NDC from plant-source foods include cellulose, hemicelluloses and pectin. Glc, Glucose; Man, Mannose; Gal, Galactose; Xyl, Xylose; Ara, Arabinose; GalA, galacturonic acid; Api, Apiose; Rha, Rhamnose; AceA, Aceric acid; Fuc, Fucose; Ac, Acetylated; Me, Methylated.

As the chemical structure strongly influences the physicochemical properties and the biological effects of NDC from plant-source foods, it is necessary to define the main structural patterns of biologically active NDC in CRC models. NDC are comprised mainly by polysaccharides from plant cell wall, such as cellulose, hemicellulose and pectin (Figure 1). Cellulose consists of relatively conserved polysaccharides with long and linear β-(*1,4*)-linked glucose (Glc) residues that vary in size according to plant species and tissue. On the other hand, hemicellulose consists of structurally complex and heterogeneous oligo- and polysaccharides with β-(*1,4*)-linked backbone of xylose (Xyl), Glc, mannose (Man), or galactose (Gal). The hemicellulosic fractions include xylans (glucoronoxylan, arabinoglucoronoxylan, arabinoxylan, and other heteroxylans), mannans (acetylated and non-acetylated mannan, galactoglucomannan, galactomannan, and glucomannan), galactans (galactan and arabinogalactan), xyloglucans, and mixed-linkage glucans (22), which vary in size and branching pattern.

Similar to hemicellulose, pectin consists of linear and ramified homo- and heteropolysaccharides; however, pectin contains relatively high amount of acidic monomers compared to hemicellulose including mainly galacturonic acid (GalA). The major fraction of pectin usually consists of linear α-(*1,4*)-linked GalA residues (homogalacturonan, HG) with varying degree of methyl and acetyl esterification. Xylogalacturonan is also a component of pectin and have the same α-(*1,4*)-linked GalA backbone as HG but substituted at C2 and C3 with β-(*1,3*)-linked Xyl residues (23). The pectic fraction also consists of branched structures named rhamnogalacturonan (RG-) I and II. RG-I is usually pointed out as the second major pectic fraction in plant-source foods and consists of a backbone of alternate α-(*1,4*)-GalA and α-(*1,2*)-rhamnose (Rha), in which the latter can be substituted at O4 mainly by arabinans, galactans, and arabinogalactans—although substitutions with Xyl and Glc residues coexist in specific plant-source foods (24). Finally, RG-II consists of an α-(*1,4*)-linked GalA backbone with complex branches made up of rare monomers (e.g., aceric acid and apiose) with different side chain, size and conformation depending on plant-food source (25–27).

As mentioned above, even though NDC is generally considered as a dietary fiber, the diversity of NDC structure from plant-source foods results in different physicochemical properties, fermentation patterns, and biological effects, thereby making the evaluation of the structure-function relationship challenging. Thus, there is an increasing number of studies exploring which specific structural patterns of NDC induce beneficial biological effects in CRC models (28–30).

## Effects of the Non-Digestible Polysaccharides on CRC Development and Progression

Studies have shown the association between the intake of specific food components and cancer, such as an inverse correlation between the intake of NDC from plant-source foods and CRC development and progression (12, 18, 31–33). However, despite the evidence that high intake of NDC could reduce the risk of CRC up to 38% (34), the levels of this evidence is still considered as probable, because of both the broad spectrum of CRC subtypes (35), and the heterogeneity of physical and biological functions of NDC from distinct plant sources (36–38). Besides that, there is the presence of others dietary components in food matrix that influence the physicochemical properties and biological effects of NDC (39). Therefore, as some studies did not consider dietary components other than NDC in plant-source foods, such as polyphenols, vitamins, and minerals (34), it is difficult to establish an inverse association between the intake of NDC and CRC risk. A reliable characterization of the complex NDC structure, followed by their isolation, purification and the study of their biological effect, is crucial to reach a desirable structure-function relationship between NDC and the anticancer effects.

**Figure 2:**
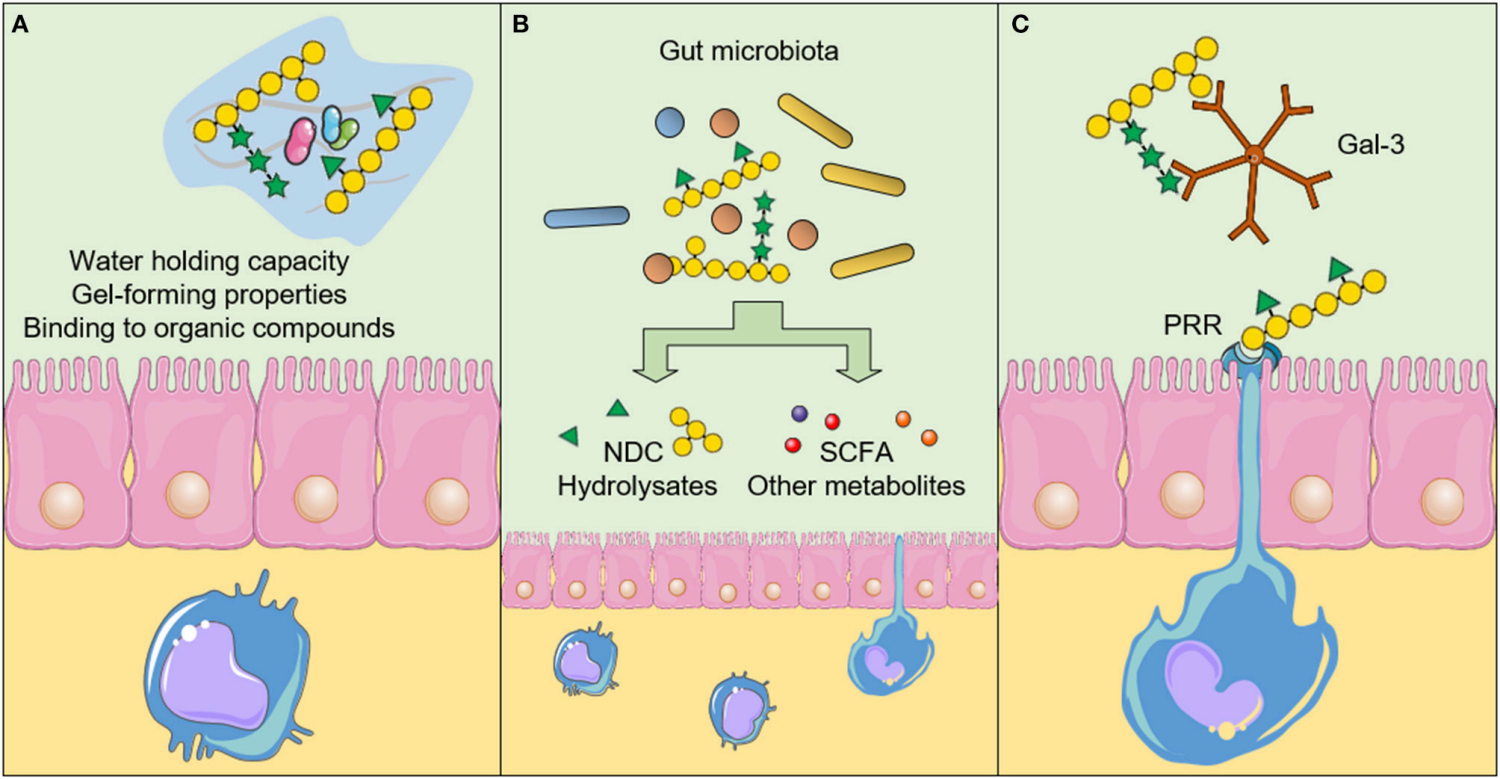
Physicochemical and biological effects of non-digestible carbohydrates (NDC) after the intake. **(A)** The physicochemical properties of NDC influence the absorption of other nutrients and reduce the interaction between carcinogens and the intestinal epithelium. Furthermore, NDC can promote an increase in satiety and stool bulk, as well as reduce the transit time throughout the gastrointestinal tract. **(B)** The fermentation-related effects result in the production of short-chain fatty acids (SCFA) and other metabolites, which can induce biological effects in epithelial intestinal cells, immune system cells and cancer cells. The fermentation of NDC by the gut microbiota can also influence the microbiota profile itself. **(C)** NDC can also interact directly with cellular components, such as the Pattern recognition receptors (PRR) and galectin-3 (Gal-3), thereby inducing downstream signaling pathways in cells and affecting cancer cell adhesion and invasion. The figure was modified from Smart Servier Medical Art (http://smart.servier.com/), licensed under a Creative Common Attribution 3.0 Unported Licens (https://creativecommons.org/licenses/by/3.0/).

There are three main mechanisms in which NDC act against CRC development and progression. The consumption of NDC can induce (A) physicochemical effects in the gastrointestinal tract, (B) fermentation-related effects, and (C) direct effects resulting from the interaction between NDC and cells, such as intestinal epithelial cells (IEC), immune system cells, and CRC cells (Figure 2). Below we summarized the physicochemical and the fermentation-related effects of NDC from plant-source foods, and focused on the recent findings that show the possible mechanisms through which distinct NDC directly interact with cells, thereby suggesting new beneficial effects regarding the intake of NDC and decreased CRC development and progression.

### Physicochemical Effects

The physicochemical effects of NDC in the gastrointestinal tract are related to the interaction of these carbohydrates with other components through gel-forming properties, water holding capacity, and the ability of binding to other organic compounds (40).

Both gel-forming properties and water holding capacity result in increased stool bulk, thereby providing satiety (41). Promising results concerning the effects of specific NDC on satiety have been stimulating industry to reformulate the nutritional composition of foods and community to change their dietary pattern, aiming to reduce obesity trends (42), which is a risk factor for CRC development (11, 43). The increase in stool bulk also contributes to the dilution of possible carcinogens. Furthermore, the reduction in stool transit time, which is a result of gastrointestinal mobility due to increased luminal content, reduces the exposition of IEC cells to carcinogens (44). NDC can also entrap other food components or metabolites, thereby influencing macronutrient digestibility or metabolite reabsorption (e.g., glucose, lipids, bile acid) (37), and having a positive impact on post-prandial insulin levels and glycaemic response. As example, β-glucan from barley, which consists mainly of linear and mixed β-(*1,4*)- and β-(*1,3*)-linked Glc polysaccharides, reduces post-prandial glycaemic response improving glycaemic control (45). Furthermore, β-glucan from distinct barley varieties bind to primary and secondary bile acids in intestine (46) and reduces bile acid reabsorption through the enterohepatic circulation, which is associated to a reduction of serum cholesterol levels (47, 48).

The abovementioned physicochemical effects of NDC are dependent on both their macrostructure (e.g., molecular weight, degree of crystallinity, and particle size) and microstructure (e.g., presence of functional groups). In terms of macrostructure, studies suggest that β-glucans from cereals should have a molecular weight above 100 kDa to increase the viscosity of the digestive effluents and to induce a positive effect on post-prandial response (49). However, oat β-glucans with lower molecular weight have also increased bile acid capacity (50). The overall structure is also a major source of variability in the physicochemical effects, since the threshold for arabinoxylans to induce a similar effect to that of β-glucans on post-prandial response is significantly lower—approximately 20 kDa (49). Furthermore, as the crystallinity of NDC influences their interaction with other components in the gastrointestinal lumen, changes in the crystallinity of NDC have a strong impact in their physicochemical effects. In this context, it was shown that rats fed with distinct celluloses with a degree of crystallinity ranging from 8 to 20% had differences in their fecal water content, which appears to be related to an inverse relationship between crystallinity and water holding capacity (51). This inverse relationship is not observed only for cellulose (52), but also for other NDC from plant-source foods including galactomannans from coconut flour (53) and fenugreek (54). The particle size of NDC also influences the physicochemical effects of NDC, as shown by the increase in water holding and lipid binding capacity of NDC from coconut after grinding (55). However, other studies demonstrated that the reduction in the particle size of NDC decreased the water holding capacity, as observed for NDC from rice bran (56), wheat bran (57), and citrus (58).

Recent studies that applied distinct processing methods (e.g., micronization, milling, and enzymatic degradation) in NDC from plant-source foods also support the relationship between changes in both the degree of crystallinity and particle size with changes in the physicochemical effects (59–61). For example, the reduction in the particle size of NDC from carrots subjected to high-pressure micronization, but not by ball milling, increases its water holding and lipid binding capacity (56).

In addition to the enzymatic- and physical-induced changes in the microstructure of NDC, studies are also exploring whether the introduction/removal of functional groups influences the physicochemical effects of specific NDC from plant-source foods. The phosphorylation of NDC from soybean does not appear to change its bile acid binding capacity. However, the water holding capacity of the phosphorylated NDC are 1.5-fold higher compared to the native NDC (62). The degree of esterification also appears to be directly related to the water holding capacity of NDC, as was found for citrus pectin (63) and more recently for NDC extracted from eggplant (64).

Therefore, processing methods that affect the macrostructure or the microstructure of NDC can be applied to control the physicochemical effects of these dietary components. The knowledge and control in NDC characteristics may in turn be useful for the selection and production of NDC from plant-source foods with desired physicochemical properties that are related to a decreased CRC risk.

### Fermentation-Related Effects

The chemical structures of NDC are crucial for colonic fermentation because not all NDC are fermented, and because different metabolites resulting from the fermentation of distinct NDC act on a broad range of downstream signaling pathways in non-cancer cells and in CRC cells (65). Besides the structure-dependent effects, the fermentation-related effects of NDC in the decrease of CRC risk are dependent of the gut microbiota itself since distinct bacteria profile will result in differentially bioactive metabolites production in a time- and structure-dependent manner (66–68).

Some bacteria from the human gut microbiota possess a large repertoire of enzymes that hydrolyse glycosidic linkages from complex carbohydrates to use the hydrolysates and some metabolites as energy sources (69). However, the identification of key bacterial species in the gut microbiota that are responsible for the disassembling of specific NDC structural patterns remains somewhat limited (25, 28, 69–72). Despite the questions that still need to be answered, the main outcomes of fermentation-related effects that contribute to a decreased CRC risk are the modulation of gut microbiota profile and the production of biologically active metabolites including short-chain fatty acids (SCFA), such as acetate, propionate and butyrate (73). The association between SCFA and the reduction of CRC risk was reviewed elsewhere (21, 74).

**Figure 3:**
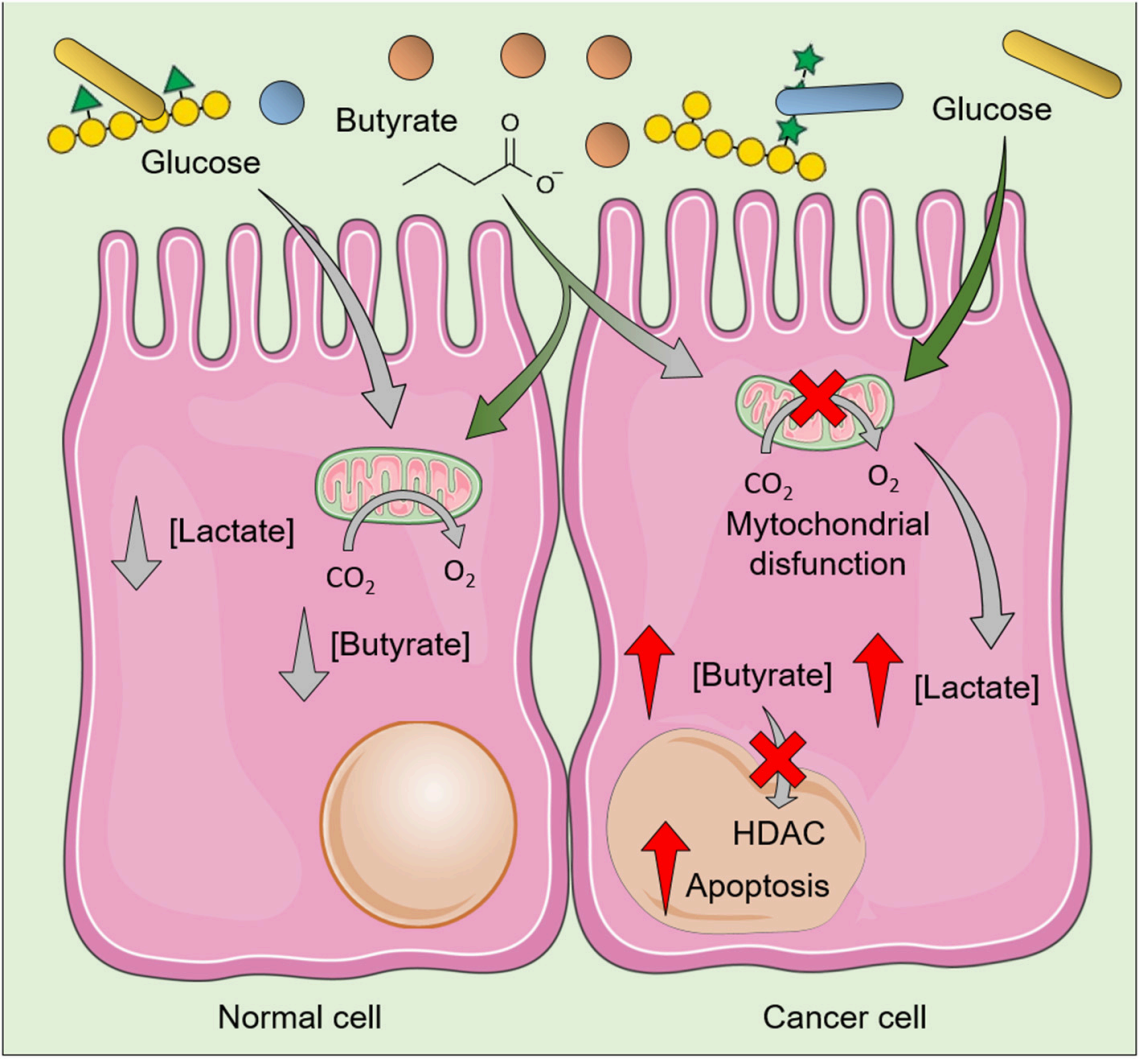
Effects of butyrate in normal cells and colorectal cancer cells (CRC). The butyrate produced during fermentation of non-digestible polysaccharides induces distinct effects in normal cell and CRC cells, as the latter rely on glucose—instead of butyrate—as their primary energy source. Increased glycolysis results in increased intracellular levels of lactate and decreased clearance/utilization of butyrate, whose increased intracellular levels inhibit histone deacetylases (HDAC) and induce death of CRC cells. As normal cells usually use butyrate as the main energy source, relatively low levels of butyrate is accumulated. The figure was modified from Smart Servier Medical Art (http://smart.servier.com/), licensed under a Creative Common Attribution 3.0 Unported Licens (https://creativecommons.org/licenses/by/3.0/).

SCFA produced after fermentation of NDC could help to maintain the lumen pH at lower levels, thereby inhibiting pathogens growth and favoring the establishment of a healthy gut microbiota. SCFA, especially butyrate, also stimulate IEC growth by functioning as the primary source of energy for these cells while being metabolized by β-oxidation in the mitochondria. Several mechanisms for SCFA uptake across the apical membrane of IEC had been proposed including transport by monocarboxylate transporter (e.g., MCT1 and SMCT1), counter-transport with bicarbonate, and passive diffusion (75). These SCFA also act in downstream signaling pathways in CRC cells (76, 77) and in non-cancer cells including IEC and immune system cells (78, 79) through interaction with G protein coupled receptors (FFAR2/GPR43, FFAR3/GPR41, GPR109a, and Olfr78) (80). Thus, the uptake of SCFA by IEC results not only in the provision of energy to normal metabolic functions but also in the production of interleukin (IL-) 18 (81), involved in the maintenance of epithelial integrity, as well as in the increased secretion of antimicrobial peptides (82). For example, butyrate reduces pro-inflammatory effects by inhibiting nuclear factor-κB (NF-κB) activation (83), as well as the Wnt signaling pathway, a pro-inflammatory pathway (84) constitutively expressed in some CRC cells (85). Besides effects in CRC cells, butyrate contributes to the normal turnover of cells in the gastrointestinal tract, as it induces proliferation of IEC at the crypt of the colon and increases apoptosis of IEC at the villus (86). Notably, this effect on proliferation does not occur at the same extent in CRC cells because cancer cells present a shift from oxidative metabolism to anaerobic glycolysis (the so-called Warburg effect), which results in the accumulation of lactic acid. As CRC cells rely on glucose as their primary energy source instead of butyrate, this shift in the metabolism of CRC cells results in accumulation of butyrate, whose increased intracellular levels inhibits histone deacetylases (HDAC), thereby resulting in cell cycle arrest and further induction of apoptosis in cancer cells (87) (Figure 3).

In addition to the induction of IL-18 by IEC, which is also crucial for intestinal immune homeostasis since IL-18 helps maintaining the balance between T helper 17 cells (T_h_17) and regulatory T cells (T_reg_) (88, 89), SCFA also interact directly with innate mechanisms of defense. In neutrophils, SCFA modulate recruitment, effector function, and cell survival (90). Phagocytes including dendritic cells and macrophages also respond to SCFA, which regulates pro-inflammatory cytokine production (91, 92).

Thus, despite CRC cells can use SCFA as energy source for proliferation (93), the fermentation-related effects on IEC and immune system cells have a clear relationship with the maintenance of host defense mechanisms and therefore with regulation of inflammatory response. As the molecular pathobiology of CRC usually implicates in pro-inflammatory conditions with an increase in the secretion of cytokines and chemokines that will promote malignant progression, invasion, and metastasis (94), the fermentation-related effects are generally regarded as an essential mechanism through which the intake of NDC relate with reduced inflammation and reduced CRC development and progression. For more detailed reviews regarding these fermentation-related effects of NDC in non-cancer and CRC cells, as well as the interplay between gut microbiota and fermentation-related beneficial effects, the reader is referred to Lam et al. (95), McNabney and Henagan (96), van der Beek et al. (97), and Zhou et al. (98).

Despite the mechanisms through which the fermentation-induced SCFA production relates to a decrease in CRC risk appear to be well-known, recent studies have also been conducted to explore how specific NDC affect gut microbiota composition. As bacterial strains have distinct prebiotic properties, changes in the microbiota composition induced by these dietary components may influence SCFA production, thereby influencing CRC risk. A previous study strongly supports this hypothesis by showing changes in the microbiota composition of children from Europe and rural Africa during the transition between breast milk feeding and the introduction of solid diet (99). The study has shown that differences between the fecal microbiota composition of children from Europe and rural Africa occurred only after the introduction of a solid diet. Children from Europe have reduced consumption of NDC from plant-source foods (e.g., arabinoxylans) compared to children from rural Africa, and this difference appears to induce an enrichment of Bacteroidetes phylum—whose members have specific genes encoding xylanases—in the gut microbiota of children from rural Africa. After this finding, numerous studies have applied *in vivo* analysis of fecal microbiota and/or *in vitro* human fecal fermentation to explore whether specific NDC induce changes in the microbiota composition, providing insights into the relationship between structure of NDC and their prebiotic function, as showed by the structure-dependent effects of NDC in promoting the survival of *Lactobacillus* spp. (100). Furthermore, a recent study (101) explored the relationship between the structure of distinct NDC from orange, lemon, lime and sugar beet, and their beneficial effects on the modulation of gut microbiota. Using an *in vitro* colonic fermentation model (TIM-2), the authors had found that the increase in the degree of esterification of HG appears to be the most important parameter in determining beneficial effects on gut microbiota composition, followed by the composition of neutral sugars (e.g., increase in HG/RG ratio and the presence of arabinose) and the reduction in the degree of branching (101).

Thus, although studies are successfully proving insights into the relationship between the structure and prebiotic function of purified NDC from plant-source foods, the preference of a specific bacterial strain in utilize an NDC from a food matrix appears to be more complex, as the fermentation rate of a single NDC is affected when others NDC and other dietary components (e.g., polyphenols) are present (39). In this context, it was found that mixing fast-fermenting NDC including HG from citrus pectin and xyloglucan from tamarind reduces their fermentation rate, thereby delaying the prebiotic effect (102). This reduction in the fermentation rate probably make NDC reach the distal parts of the colon, which is of particular importance in terms of CRC risk. The reduced fermentation rate in the distal part of the colon have been thought as one of the reasons why most of CRC are detected in this region. This delay in the prebiotic effect by mixing different NDC appear to occur because of the hierarchical preference, which refers to the ability of a bacterial strain in prioritize the utilization of some NDC before others (103). Studies confirm the hierarchical preference by observing that a bacterial strain can prioritize the fermentation of specific host mucosal glycans (104) or NDC from plant-source foods (105). Examples of hierarchical preference include the preference of *Bacteroides thetaiotaomicron* in utilizing galactan from potato instead of arabinan from sugar beet (103), as well as the increased ability of *Bacteroides* spp., *Bifdobacterium* spp., *Faecalibacterium* spp., and *Lactobacillus* spp. in utilize fructans with low molecular weight compared to fructans with high molecular weight (106, 107). These hierarchical preferences appear to be strain-specific, as closely related gut bacterial strains (e.g., *B. thetaiotaomicron* and *B. ovatus*, or *L. delbruckii* and *L. paracasei*) prioritize the use of distinct NDC (71, 108). Furthermore, recent studies are focusing on evaluating whether distinct gut microbiota profiles can utilize specific NDC from plant-source foods, such as arabinoxylans from corn and sorghum, and fructans from chicory root (105, 109). Therefore, studies using more complex samples such as the whole food or a mix of NDC instead of using a single NDC, as well as studies comparing the ability of distinct microbiota profiles in utilizing the same NDC—as performed by Yang et al. (110), Chen et al. (105) and Brahma et al. (111)—are elucidating practical knowledge required to use prebiotic therapy or diet modifications to benefit the function of specific bacterial strains that relates to a decreased CRC risk.

### Direct Effects

NDC share structural features to lipopolysaccharides and other structural carbohydrate-containing molecules at the surface of bacteria (112, 113). As these carbohydrates from bacteria directly interact with IEC and immune system cells along the gastrointestinal tract, it was hypothesized that NDC from plant-source foods also directly interact with cells in the gut.

The abovementioned hypothesis has been confirmed through *in vitro* studies and most recently through *in vivo* studies (114). Since pattern-recognition receptors (PRR) in cells are the main responsible for the recognition of bacterial carbohydrates, efforts have been made mainly on the investigation of the PRR-mediated effects of NDC (115), although some direct but PRR-independent mechanisms have also been described (116, 117) and will be pointed out later in this review.

#### Pattern Recognition Receptor-Mediated Effects

PRR existing in IEC and immune system cells regulates epithelial proliferation and intestinal permeability, and maintains gut homeostasis through recognition of harmful organisms and endogenous metabolites (118). Furthermore, PRR plays an important role in shaping intestinal microbiota in both composition and number by interacting with commensal bacteria (119). Thus, PRR-mediated signaling pathways result in immune surveillance and in maintenance of host-bacteria interaction alongside the gastrointestinal tract, whose dysregulation is clearly associated with increased CRC risk (98, 120). As NDC influence microbiota profile and therefore the formation of specific metabolites in the gut lumen, the intake of plant-source foods rich in NDC influences the PRR-mediated responses through an indirect mechanism. Interestingly, NDC also directly interacts with PRR in a structure-dependent manner (114, 115).

The PRR include RNA helicases (RLR), Nucleotide binding oligomerization domain (NOD)-like receptors (NLR), C-type lectin receptors (CLR), and Toll-like receptors (TLR), which recognize distinct evolutionarily conserved pathogen-associated molecular patterns (PAMP) of microorganisms—such as the carbohydrate-containing molecules at the surface of microorganisms—as well as endogenous damaged-associated molecular patterns (DAMP) (120). Despite the variety of PRR in human cells, only few PRR has been shown to recognize NDC from plant-source foods (Table 1). Most of studies focused in the interaction between NDP from plant-source foods and TLR, but we will also describe published studies that report the effects of NDC from foods through NLR- and CLR-dependent pathways.

**Table 1:** Pattern-recognition receptors (PRR) that recognizes non-digestible carbohydrates (NDC) from plant-source foods.

**PRR**	**NDC**	**Plant-source food**	**Effects**	**References**
**NLR**				
NOD2	Inulin	Chicory root	Activation in HEK cells, NF-κB release	(115)
NLRP3	HG, RG-II and HC	Chayote	Inhibition of NLRP3 priming in macrophage-like cells	(121)
**CLR**				
Dectin-1	Mixed linkage β-glucan	Barley	Activation in immune cells, NF-κB release, IL-6 and IL-8 release	(122)
Dectin-1	Arabinoxylan	Wheat	Inhibition in HEK cells	(123)
**TLR**				
TLR2	Inulin	Chicory	Activation in THP-1 cells, NF-κB release	(115)
TLR2	RS2	Maize	Activation in HEK cells, NF-κB release	(124)
TLR2	Maltooligosaccharides	Wheatgrass	Activation in immune cells	(125)
TLR2 and 4	FOS	Rice	Induction of dendritic cell maturation in mice	(126)
TLR2 and 4	HG (varying degree of ME)	Lemon	Activation in T84 cells, maintenance of intestinal epithelial barrier integrity	(127)
TLR2 and 5	RS3	Maize	Activation in HEK cells, NF-κB release	(124)
TLR4	Galactan	Apple	Inhibition of LPS-induced activation in a colitis model	(128)
TLR4	HG (varying degree of ME)	Citrus	Inhibition of LPS-induced activation in a colitis model	(129, 130)
TLR4	HG (branched)	Citrus	Inhibition in immune cells	(129)
TLR4	Levan	Soybean	Cytokine release in mice	(131)
TLR4-8	Inulin	Chicory	Activation in THP-1 cells, NF-κB release	(115)
**HETERODIMERS**				
TLR1\TLR2	HG (low degree of ME)	Lemon	Inhibition of intestinal inflammation	(132)
Dectin-1\TLR2	Galactomannan	Guar gum	Inhibition of IEC *in vitro* and in a colitis model	(133)
Dectin-1\TLR2	Galactomannan	Guar gum	Inhibition of IEC in a colitis model	(134)

*NLR, nod-like receptors; CLR, C-type lectin receptors; TLR, tool-like receptors; HG, homogalacturonan; RG-II, rhamnogalacturonan II; AG, type II arabinogalactan; ME, methyl esterification; RS, resistant starch; FOS, fructooligosaccharides*.

#### Nucleotide Binding Oligomerization Domain-Like Receptor

All NLR have C-terminal leucine-rich repeat motifs (LRR) for ligand sensing, except NLRP10 (135), and a NACHT domain that facilitates protein oligomerization (136). The presence of the LRR motif and NACHT domain are essential for NLR function, which acts as a scaffolding cytosolic protein that assembles signaling platforms triggering NF-κB and mitogen-activated protein kinase (MAPK) signaling pathways, thereby controlling the activation of several caspases (136). Thus, the activation of NLR usually results in the assembling of pro-inflammatory complexes termed inflammasomes, which relates not only to NF-κB- and MAPK-mediated secretion of cytokines and chemokines but also to the activation of a myriad of cell death regulators (137). Therefore, the dysregulation of NLR have been associated with infections, autoimmune diseases, inflammatory disorders and cancer, such as CRC (138, 139).

Among the more than 20 NLR that have been identified in human cells (140), NOD1, NOD2 and NLRP3 are pointed out as the most important in terms of relevant biological functions and CRC development (137). The mechanisms through NLR-mediated effects are associated with increased CRC risk related mainly to an excessive NLR-induced chronic pro-inflammatory microenvironment (141). Intriguingly, NLR agonists, specially NOD1 and NOD2 agonists, have been proposed as therapeutic agents in CRC treatment because both experimental studies showed that NOD1 deficiency leads to increased tumorigenesis in mice (142) and the activation of these NLR may regulate the pro-inflammatory effects induced by other PRR (143, 144). Thus, although both beneficial and deleterious effects of NLR activation on CRC remain not fully understood, it seems that punctual activation or inhibition of NLR could differentially induce effects during cancer development, progression, and metastasis.

Despite the relevance of NLR in CRC risk, few studies have focused on exploring the effects of NDC from plant-source foods in the regulation of NLR because they are cytosolic receptors. Phagocytes including macrophages can internalize NDC (145), but the exact mechanism of interaction between NDC and cytosolic receptors is not proven. Furthermore, it is known that IEC can transfer exosome-like vesicles from their apical or basolateral surface to both mesenteric lymph node and gut lumen (146), and these cells can also take up exosomes from foods (e.g., bovine milk exosomes) or produced by immune system cells (147). However, despite these evidences, it is not possible to assert that vesicle trafficking between cells—or between cells and food components—is responsible for the internalization of NDC. Despite the lack of evidences on the mechanisms through which NDC interact with cytosolic receptors, linear β-(*1,2*)-linked fructose oligosaccharides (inulin) from chicory root also activate NOD2 in HEK 293 hNOD2 reporter cells (115).

Furthermore, NDC from chayote fruit, which consists mainly of pectic homogalacturonan and highly branched RG-II, as well as hemicellulosic material including glucomannan, xyloglucan, and glucurono(arabino)xylan, inhibits NLRP3 inflammasome activation in human THP-1 macrophage-like cells. The effects of this NDC on NLRP3 inflammasome can be considered an indirect effect of the interaction between the NDC and other PRR that are essential to induce priming signals required for NLRP3 inflammasome activation (121). In fact, cross-talk between PRR may occur through three main mechanisms. It can be a (a) requirement of two or more PRR for a specific biological response, (b) an interaction between PRR for robustness or redundancy of biological response, or (c) a negative regulation between PRR (148). Thus, although the effects of NDC from chayote were not directly explored in CRC cells, it can be proposed that NDC-mediated effects on the modulation of cytosolic NLR may occur through indirect mechanisms that involve the interaction between NDP and other PRR. Therefore, despite the investigation of NDC-induced effects on NLR is important to gain further insights into the biological effects induced by NDC from plant source foods, further analyses are necessary to explore the interaction between the NDC and other PRR, such as CLR and TLR.

##### C-type lectin receptors

Unlike NLR that are cytosolic proteins, the main CLR (Dectin-1, Dectin-2, Mannose receptor, and macrophage inducible Ca^2+^-dependent lectin—Mincle) are trans-membrane PRR widely expressed in myeloid cells. Glycosylated structures are the natural ligands of CLR, which contain conserved carbohydrate-recognition domains (CRD). Thus, it is easy to think that some food-derived carbohydrates interact with CLR. In this regard, studies had explored the interaction between NDC from foods and CLR, especially Dectin-1. However, the effects were investigated mainly using fungal-source foods (149–151) compared to plant-source foods (122, 133). As CLR is expressed mainly in antigen-presenting cells including macrophages and dendritic cells, the study of the interaction between NDC from foods and CLR focused mainly on innate immune responses against pathogens and cancer, including CRC.

Activation of CLR can induce anti-inflammatory effects, as observed by the activation of the heterodimer Dectin-1\TLR2, which increases suppressor of cytokine signaling (SOCS)-1 expression, thereby resulting in anti-inflammatory effects (152). However, in general, the inhibition of CLR in some CRC cells (153) promotes cell apoptosis (154) and suppresses a pro-inflammatory phenotype, thereby reducing CRC risk (155). As CLR also facilitates adhesion of head, neck and breast cancer cells to the lymphatic endothelium and thus favor tumor invasiveness (156), NDC-mediated inhibition of CLR may directly impact in the invasiveness of cancer cells. On the other hand, CLR is essential for the recognition of altered glycosylated membrane proteins of CRC cells by immune system cells (157, 158). Thus, as NDC-mediated inhibition is beneficial in cancer cells but may suppress anticancer response by the innate immune system, further studies using *in vivo* models may elucidate possible benefits on the interaction between NDC from plant-source foods and CLR in decreasing CRC risk.

Among all CLR, Dectin-1 seems to have a major impact in innate immune responses against cancer and are present in CRC cells (153, 157). Dectin-1 is a specific receptor for β-glucan (159, 160), which is the most abundant fungal cell wall polysaccharide—and is also a constituent of the bacterial cell wall (161, 162). As β-glucan is a naturally occurring NDC in mushrooms and some plant-source foods, especially in cereal grains (163), Dectin-1 is by far the most studied CLR in terms of interaction with food-derived NDC. Furthermore, as Dectin-1 expression is high in phagocytes such as macrophages and dendritic cells, β-glucan from foods seems to act first through interaction with these innate immune system cells (164).

Phagocytes can extrude their dendrites across the intestinal epithelium into the gastrointestinal lumen and diet-derived β-glucan could interact with them through Dectin-1. Upon activation of Dectin-1, β-glucan induces mainly the Spleen tyrosine kinase (Syk)-dependent pathway, which triggers adaptive immune response in T cells and B cells that results in the inhibition of both tumor growth and metastasis (165). Despite the inhibition of Dectin-1 induces apoptosis in CRC cells *in vitro* (154), evidence that showed the positive role of Dectin-1 activation in the decrease of CRC risk comes from the findings that a loss of function of this CLR is associated with increased risk of ulcerative colitis (166). Furthermore, the relationship between the loss of Dectin-1 function due to polymorphism and the increasing risk of inflammatory disorders in the gastrointestinal tract (158), as well as other *in vivo* studies (167, 168), supports the beneficial effects of Dectin-1 activation in the reduction of CRC risk.

In this context, a barley-derived β-glucan that consists of linear and mixed β-(*1,4*)- and β-(*1,3*)-linked Glc residues interacts with Dectin-1 and triggers a Syk-dependent pathway that results in the activation of NF-κB of immune system cells, leading to cytokine secretion including IL-6 and IL-8 (122). Thus, it is possible that some NDC from plant-source foods, especially β-glucans, directly activate Dectin-1 and induce positive effects in the reduction of CRC risk. In addition to enhancing the innate immune response, CRL seems to function together with other PRR, especially with TLR (151, 169, 170), to regulate the function of IEC. In this context, a recent study showed that guar gum exerted *in vitro* and *in vivo* anti-inflammatory effects in IEC through a Dectin-1\TLR2-dependent pathway (133). The same mechanism seemed to be related to the anti-inflammatory effects of partially hydrolysed guar gum—which consists of a backbone containing β-(*1,4*)-linked Man residues and short branches containing Gal at C4—in a colitis model (134).

##### Toll-like receptors (TLR)

TLR are the most studied class of PRR because of both the variety of PAMP and DAMP that interact with these germline-encoded PRR and also because of the biological outcomes that TLR-induction/inhibition could cause in human health (171). Currently, 13 TLR have been identified in human cells, among intracellular and extracellular receptors (172). As NLR, TLR are evolutionary conserved PRR-containing LRR motifs for ligand sensing that induces NF-κB and MAPK signaling. Most of TLR activate NF-κB and MAPK mainly through the adaptor myeloid differentiation factor 88 (MyD88), except TLR3, which triggers NF-κB and MAPK through a TIR-domain-containing adapter-inducing interferon-β (TRIF)-dependent pathway (173).

Among all TLR, TLR2 and TLR4 have been the most studied ones concerning the interaction with NDC from plant-food sources (115, 127). TLR2 recognizes different kinds of PAMP including lipoprotein, lipoteichoic acid, and peptidoglycan molecules from Gram-positive bacteria, as well as DAMP, such as heat shock proteins (HSP). TLR4 interacts with lipopolysaccharide (LPS) from Gram-negative bacteria and with several DAMP including HSP, fibronectin, and heparan sulfate (174).

In the context of CRC, several studies had explored the role of TLR on cancer development, progression, and invasion, as reviewed by Li et al. (175). Furthermore, results of a recent prospective cohort study suggested that the protective effect of NDC on CRC risk may involve interactions between the NDC and TLR4, and that polymorphisms in TLR2 and TLR4 are associated with increased CRC risk (176). In fact, CRC development and progression have been correlated with TLR2 and TLR4 overexpression in CRC (177). CRC cells express both mutated TLR2 and MyD88, thereby resulting in increased activation of TLR2-dependent pathways. Thus, TLR2 inhibitors were proposed as therapeutic agent in CRC (178).

TLR4 is considered the most important inflammatory inducer amongst all TLR, thereby playing a key role in immune response against intestinal pathogens. However, excessive activation of TLR4 may enhance not only immune response but also gives rise to cancer progression through disruption of intestinal immune homeostasis (179). Excessive TLR4 activation also induces macrophages apoptosis after activation of the receptor-interacting serine/threonine-protein kinase (RIPK) 1 and RIPK3, which induces cell lysis and necroptotic death (180). Furthermore, enhanced expression of TLR4 in CRC cells promotes cell survival, epithelial-mesenchymal transition (181), and downregulates the expression of the death receptor Fas in cancer cells (182, 183). Thus, excessive activation of TLR4 increases the risk of inflammatory diseases and CRC, and—as occur to TLR2—potential inhibitors of TLR4/NF-κB pathway have also been considered as therapeutic agents in CRC (184).

Despite the abovementioned evidences show that inhibition of TLR4-dependent signaling pathways may reduce CRC risk, some NDC from plant-source foods including citrus pectin and ginseng polysaccharides have potential anticancer effects that seems to be related to TLR4-mediated activation (127, 185–187). The most reasonable explanation is that these NDC from plant-source foods do not activate TLR4 at the same extent as natural PAMP, such as LPS. Therefore, NDC-induced TLR4 activation reduces the interaction of this PRR with more potent ligands (186–188). Furthermore, TLR-mediated activation can launch a strong immune response to assist cancer treatments and/or activate TLR-dependent programmed cell death, including apoptosis, autophagy, and necroptosis (189).

Apart from the biological relevance of TLR2 and TLR4, TLR3 activation with polyinosinic:polycytidylic acid induced apoptosis of in CRC cells (190), whereas TLR5 activation suppressed CRC growth and induced necrosis of cancer cells *in vivo* (191). Furthermore, TLR9-induced activation in immune system cells promoted cell survival and therefore enhanced immune response against cancer; however, the role of TLR9 in CRC remains unclear (175), as CRC cells have reduced expression of TLR9, suggesting a protective role of TLR9 expression against malignant transformation in the gastrointestinal tract (192).

Molecules from plant-source foods including polyphenols (193, 194) and NDC have been found to exert effects on TLR-mediated pathways (114, 115, 132). In the context of NDC from plant-source foods, it was found that apple galactan suppressed LPS-induced activation of TLR4 downstream signaling in an *in vivo* model of colitis-induced CRC (128). Furthermore, NDC from apple reduced the migration of CRC cells *in vitro* (195), and enhanced the inhibitory effect of 5-fluorouracil in the growth of CRC cells (196). Homogalacturonan-rich fractions from lemon pectin with varying degree of methyl esterification also induced TLR2- and TLR4-mediated responses (127). Authors have showed that the varied degree of methyl esterification in the homogalacturonan residues of lemon pectin strongly influenced TLR2-mediated responses, but did not affect TLR4-mediated response. Furthermore, both low- and high-methoxylated lemon pectin seemed to exert positive effects on the maintenance of intestinal epithelial barrier integrity *in vitro* (127). Recently, it was found that the inhibitory effect of low-methoxylated pectin from lemon suppressed the pro-inflammatory TLR2\TLR1 pathway while the heterodimerization between TLR2 and TLR6, which induces a tolerogenic effect, was not induced by lemon pectin (132). Furthermore, authors found that the administration of low-methoxylated pectin from lemon prevented intestinal inflammation *in vivo* in a fermentation-independent manner. Notably, similar TLR-inhibitory effects are in agreement with *in vivo* studies using low- and high-methoxylated citrus pectin, which attenuated both endotoxin shock through a TLR-dependent pathway (129), as well as inflammatory effects in a colitis model (130).

In addition to the TLR-mediated effects of NDC from apple and citrus, it was found that inulin from chicory roots with distinct chain-lengths interacted not only with TLR4, but also with TLR5, TLR6, TLR7, and TLR8 in a MyD88-dependent pathway, and had no effects on cytosolic TLR3 and TLR9 (115). Although it is not clear the biological outcome resulted from the interaction between these NDC and TLR, the observation of TLR-mediated effects by NDC from plant-source foods may support further studies aiming to evaluate the direct effects of these dietary components in CRC development and progression.

#### Direct Interaction With Galectin-3

In addition to the direct effects of NDC from plant-food sources through PRR-dependent mechanisms in IEC, immune system cells and CRC cells, NDC can also directly interact with cellular components in a PRR-independent pathway. The main PRR-independent effect seems to be related with the interaction between NDC from plant-source foods and the galectin-3 (Gal-3).

Gal-3 is a protein of the lectin family that has a CRD with strong affinity for β-galactosides. Notably, it has been consistently shown in the past two decades a strong association between increased levels of Gal-3 and several types of cancer including CRC (197, 198). Gal-3 expression had been correlated not only with CRC incidence, but also with CRC severity, as increased levels of Gal-3 are associated with a worse cancer prognostic (197–199). A recent study supported this correlation by showing that the positive expression rate of Gal-3 in CRC tissues is approximately 5-fold higher compared to cancer-adjacent tissues (198). Furthermore, authors suggest a direct association between positive expression of Gal-3 and both tumor size and malignant progression.

Gal-3 is present intracellularly—at the cytoplasm or within the nucleus—attached to cell surface, or in the extracellular media as a dimer or as a pentamer (200). Regardless the localization of Gal-3, the increase in its levels is related with increased CRC risk and severity because this protein is involved in a wide range of cancer-promoting effects including CRC cells adhesion, invasiveness, growth, and proliferation (201, 202). Notably, some NDC from plant-source foods bind to the CRD of Gal-3 and therefore inhibits Gal-3-mediated effects, which includes not only attachment to glycan-containing surfaces (203), but also with downstream signaling mechanisms that inhibits both apoptosis and cell cycle arrest in CRC cells (204). Furthermore, Gal-3 appears to be associated to multiples mechanisms related to chemo-resistance of CRC cells by enhancing drug efflux, DNA repair mechanisms and activating signaling pathways (e.g., Wnt, Hedgehog and Notch) associated with multi-drug resistance (205). Thus, since the observation of specific bind of NDC from plant-source foods to Gal-3 (206), several studies have been performed to assess the interaction between distinct NDC and Gal-3, as well as the effects of this interaction in CRC progression, as described previously (117).

Among NDC from plant-food sources and Gal-3 inhibition, the modified citrus pectin (MCP) is the most studied one (185, 207–209). MCP is a preparation derived from citrus pectin that is modified by high temperature, alteration of pH and/or pectinase treatment, which result in the partial hydrolysis of glycosidic linkages, thereby generating smaller and less ramified NDC structure. These processes of MCP modification release neutral chains of galactan with high affinity to the CRD of Gal-3 (206, 207), which induce a broad range of inhibitory effects that had been extensively studied through *in vitro* and *in vivo* studies (207, 210, 211).

Modified sugar beet pectin, papaya pectin, and ginseng pectin have structures composed of neutral (*1,4*)-β-galactose residues, which were related to Gal-3 interaction and inhibition (208, 209, 212, 213). As observed for MCP, the binding between these pectin and Gal-3 has been associated with both *in vitro* and *in vivo* effects on CRC (209, 214). Besides the interaction with Gal-3, molecular size-fractionated MCP showed other effects than inhibit Gal-3, as leading CRC cells to apoptosis and inhibiting their migration (215). NDC from plant-source food also seem to interact with Gal-3 through non-specific binding as suggested by previous study (216). Since homogalacturonans contain relatively high amounts of charged GalA residues, it is possible that charge-charge interactions between GalA residues and Gal-3 induce a non-specific binding that may exert inhibitory effects on Gal-3 activity. Recently, it was also shown that NDC from plant-source foods can interact with Gal-3 by a combination of homogalacturonan and RG residues acting in concert, as the homogalacturonan seem to interact with RG exposing additional galectin-binding sites of the NDC, thereby enhancing Gal-3-binding properties (217).

As NDC are essentially polyhydroxy molecules, which are often esterified, it is possible that the NDC from plant-source foods interacts with other cellular components. The studies that had shown anticancer effects of NDC through interaction with Gal-3 support further studies aiming the investigation of the interaction between NDC and other signaling mediators related to the decreased CRC risk.

## Concluding Remarks

**Figure 4:**
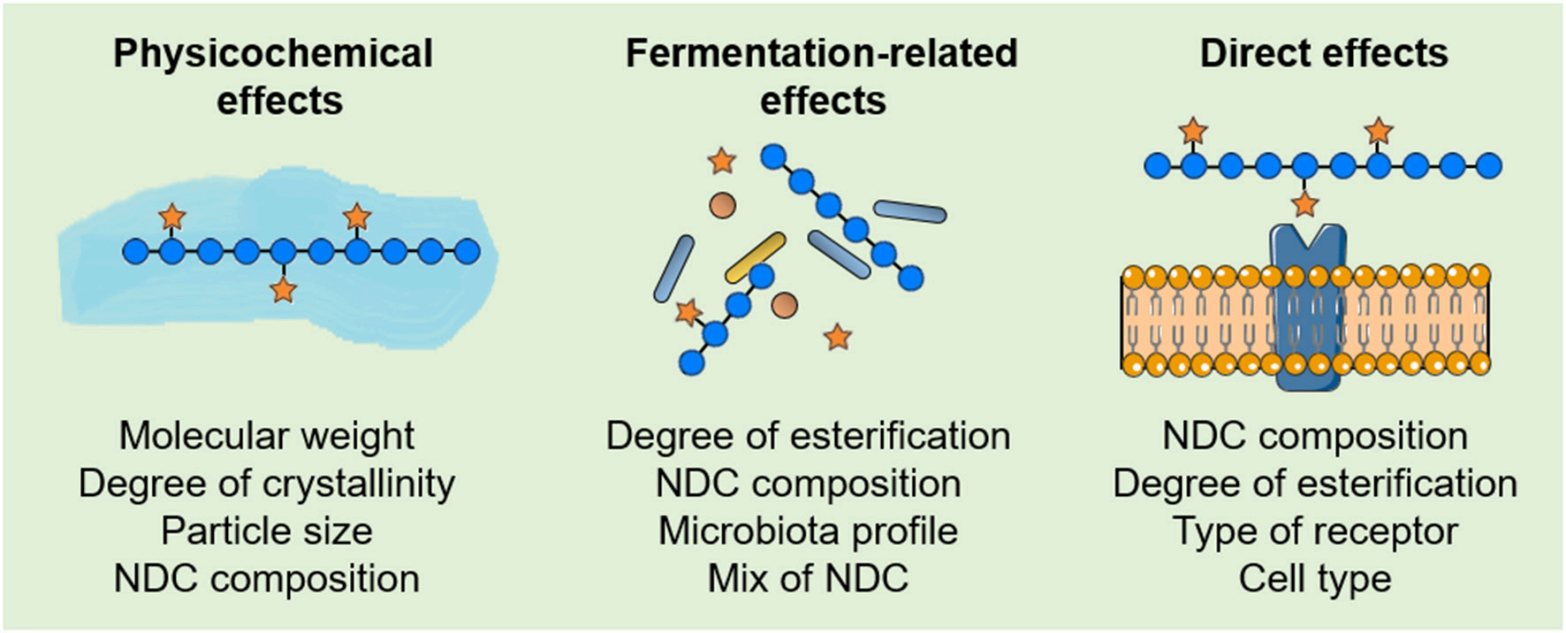
Features that influence the effects of non-digestible carbohydrates (NDC) from plant-source foods in colorectal cancer (CRC). Some of the intrinsic properties of NDC, as well as individual characteristics among cells and individuals, that influence the physicochemical, fermentation-related and direct effects of NDC from plant-source foods on the reduction of CRC risk.

The complexity of biological effects resulting from the intake of NDC from plant-source foods and their relationship with decreased CRC risk can be divided into physicochemical effects, fermentation-related effects, and PRR-dependent and PRR-independent direct effects. However, in biological systems, these complex NDC effects occur at the same time in an intricate—and poorly understood—relationship. Although the evaluation of a specific biological effect does not fully answer whether a single NDP from a plant-source food relates to a decreased CRC risk, it can provide further insights to elucidate the structure-function relationship between NDC and their effects in CRC development and progression. Therefore, as recent studies are demonstrating that intrinsic properties of NDC from plant-source foods, as well as individual characteristics among cells and individuals, strongly influence the beneficial effects of NDC on the reduction of CRC risk (Figure 4), similarities between the intrinsic properties of NDC from distinct plant-source foods may drive the discovery of new bioactive NDC. Clearly, studies that integrate the structural characterization of NDC from plant-source foods with their physicochemical, fermentation-related, and/or direct effects will provide insights not only for a better understating of the structure-function relationship between the intake of NDC and CRC risk but also for improving nutritional recommendations of NDC for healthy individuals, as well as CRC patients.

## Author Contributions

VC-A, SP, and GF wrote the manuscript. JF critical review of the manuscript. SP and VC-A illustrations. VC-A, GF, SP, and JF revised and approved the manuscript.

### Conflict of Interest Statement

The authors declare that the research was conducted in the absence of any commercial or financial relationships that could be construed as a potential conflict of interest.

## Abbreviations

AceA: aceric acid
Ac: acetylated
Api: Apiose
Ara: Arabinose
CRD: Carbohydrate recognition domain
CLR: C-type lectin receptors
CRC: Colorectal cancer
DAMP: damaged-associated molecular patterns
Fuc: fucose
Gal: galactose
GalA: galacturonic acid
Gal-3: Galectin-3
Glc: Glucose
HSP: heat shock proteins
HDAC: hystone deacetylases
HG: homogalacturonan
IEC: intestinal ephitelial cells
LPS: lipopolysaccharide
LRR: C-terminal leucine-rich repeat motif
Man: mannose
MAPK: mitogen-activated protein kinase
Me: methylated
MCP: modified citrus pectin
NDC: non-digestible carbohydrates
NRL: nucleotide binding oligomerization domain (NOD)-like receptors
PAMP: pathogen-associated molecular patterns
PRR: pattern recognition receptors
RIPK: receptor-interacting serine/threonine-protein kinase
RG: rhamnogalacturonan
Rha: rhamnose
SCFA: short-chain fatty acids
TLR: toll-like receptors
Xyl: xylose.
